# Maternal Hypoxia Increases the Excitability of Neurons in the Entorhinal Cortex and Dorsal Hippocampus of Rat Offspring

**DOI:** 10.3389/fnins.2022.867120

**Published:** 2022-04-12

**Authors:** Dmitry V. Amakhin, Elena B. Soboleva, Tatiana Yu. Postnikova, Natalia L. Tumanova, Nadezhda M. Dubrovskaya, Daria S. Kalinina, Dmitrii S. Vasilev, Aleksey V. Zaitsev

**Affiliations:** ^1^Sechenov Institute of Evolutionary Physiology and Biochemistry, Russian Academy of Sciences, St. Petersburg, Russia; ^2^Institute of Translational Biomedicine, Saint Petersburg State University, St. Petersburg, Russia

**Keywords:** prenatal hypoxia, dosed electroconvulsive shock, whole-cell patch-clamp, entorhinal cortex, hippocampus, rat

## Abstract

Prenatal hypoxia is a widespread condition that causes various disturbances in later life, including aberrant central nervous system development, abnormalities in EEG rhythms, and susceptibility to seizures. Hypoxia in rats on the 14th day of embryogenesis (E14) disrupts cortical neuroblast radial migration, mainly affecting the progenitors of cortical glutamatergic neurons but not GABAergic interneurons or hippocampal neurons. Thus, hypoxia at this time point might affect the development of the neocortex to a greater extent than the hippocampus. In the present study, we investigated the long-term effects of hypoxia on the properties of the pyramidal neurons in the hippocampus and entorhinal cortex (EC) in 3-week-old rats subjected to hypoxia on E14. We observed a reduction in the total number of NeuN-positive neurons in EC but not in the CA1 field of the hippocampus, indicating an increased cell loss in EC. However, the principal neuron electrophysiological characteristics were altered in the EC and hippocampus of animals exposed to hypoxia. The whole-cell patch-clamp recordings revealed a similar increase in input resistance in neurons from the hippocampus and EC. However, the resting membrane potential was increased in the EC neurons only. The recordings of field postsynaptic potentials (fPSPs) in the CA1 hippocampal area showed that both the threshold currents inducing fPSPs and population spikes were lower in hypoxic animals compared to age-matched controls. Using the dosed electroshock paradigm, we found that seizure thresholds were lower in the hypoxic group. Thus, the obtained results suggest that maternal hypoxia during the generation of the pyramidal cortical neurons leads to the increased excitability of neuronal circuitries in the brain of young rats. The increased excitability can be attributed to the changes in intrinsic neuronal properties.

## Introduction

The action of various pathological factors (hypoxia, radiation, acute, and chronic stress) on the maternal organism during gestation is a common cause of developmental disorders and neurological diseases, including epilepsy (Sadaghiani and Saboory, 2010; Spear, 2013; Nalivaeva et al., 2018). During embryogenesis, there are critical periods when the vulnerability of the developing central nervous system to these factors is higher, and the consequences of hypoxia are most significant (Rice and Barone, 2000). These early embryonic critical periods of brain development are associated with neurogenesis and neuroblast migration into various brain divisions (Ang et al., 2006; Golan et al., 2009; Vasilev et al., 2016). The disturbances of the radial migration of the neuroblasts into the cortical plate lead to various abnormalities in brain function, which can manifest themselves in later life. Furthermore, the disturbing cortical cell migration after hypoxia leads to the long-term consequences described in the literature—some motor and cognitive dysfunctions associated with the neuronal cells death in the postnatal period (Zechel et al., 2005; Golan et al., 2009; Stevens et al., 2013; Vasilev et al., 2016). Continuous hypoxic conditions during brain development affect both radial (Zechel et al., 2005) and tangential (Stevens et al., 2013) migration of cortical neuroblasts, causing changes in the cortical plate structure in newborn rats. The pyramidal neurons in the hippocampus are vulnerable to prenatal hypoxia during the period of the radial neuroblast migration (Golan et al., 2009). However, the critical periods for the cerebral cortex and hippocampus are different (Rice and Barone, 2000).

The hypoxia on 14th day of embryogenesis (E14) leads to multiple behavioral consequences including learning and memory impairments (see Golan and Huleihel, 2006 for the reviews) suggesting this period might be critical for brain structures involved in memory functions. We showed earlier that besides alterations in cortical morphology and functions, hypoxia on E14 affects long-term potentiation (LTP) and the density of the synapses in the CA1 hippocampal area (Zhuravin et al., 2019). In addition, the hypoxia on E14–16 decreased the neuronal cell number in the CA1 area and impaired spatial long-term memory in the Morris water navigation task (Vetrovoy et al., 2021). Therefore, E14 is within the critical period for both brain cortex areas and dorsal hippocampus in rats. The generation of cortical projection pyramidal neurons in mammals precedes the generation of inhibitory interneurons (Stevens et al., 2013), and the early part of this period (E11–E15 in rats) was shown to be crucial for the cortical cytoarchitecture (Vasilev et al., 2016). Therefore, we hypothesize that hypoxia during the critical periods of embryogenesis might disturb the balance of the excitation and inhibition in the cerebral cortex and hippocampus.

Hypoxia leads to cognitive impairments in animals (Nyakas et al., 1996; Zhuravin et al., 2019), alters EEG rhythm carachteristic (Kalinina et al., 2019), and increases vulnerability to proconvulsive drugs (Sadaghiani and Saboory, 2010; Kalinina et al., 2019). However, to our knowledge, no reports have been published concerning the changes in biophysical properties of hippocampal and cortical neurons in animals subjected to hypoxia during embryogenesis.

The early period of neuroblast radial migration into the cortical plate occurs on the 13–14th day of embryogenesis (E14) in rats, and it is crucial for a proper architecture of the neocortex and neuronal network function in later life (Caviness and Rakic, 1978; Zhuravin et al., 2009, 2019; Vasilev et al., 2016). Previously, we have shown that hypoxia on E14 affects the formation of cortical cytoarchitecture and neuronal plasticity and alters behavior during postnatal ontogenesis. On the other hand, hypoxia on E18 does not affect the cortical structure and parietal cortex-dependent behavioral tasks (Vasilev et al., 2016).

Animals subjected to prenatal hypoxia exhibit alterations in subunit composition of NMDA receptors in the CA1 area of the hippocampus (Zhuravin et al., 2019; Vetrovoy et al., 2021), and an upregulated glutamate uptake system (Kalinina et al., 2019). The effect of prenatal hypoxia on the cholinergic system in brain cortical structures was also shown (Kochkina et al., 2015; Morozova et al., 2020).

In our previous study, we have found that rats subjected to prenatal hypoxia display altered frequency characteristics of the electrocorticogram power spectrum density during slow-wave sleep and wakefulness, as well as longer episodes of 4-aminopyridine-induced spike-wave discharges compared to the controls (Kalinina et al., 2019), which can be explained by the changes in the balance of the excitation and inhibition in the cortical networks. We also found an increased expression of the glutamate excitatory amino acid transporter 1 (EAAT1) in the parietal cortex of hypoxic rats but not in the hippocampus (Kalinina et al., 2019). The observed differences in electrocorticographic rhythms in hypoxic rats might be produced both by the increased glutamate exocytosis, which leads to increased activity of its reuptake (Doi et al., 2009), and by the increased excitability of cortical neurons. Based on these observations, we hypothesize that prenatal hypoxia may lead to the increased excitability of cortical and hippocampus neurons.

The present study examined the long-term effects of maternal hypoxia on E14 (shown to be critical for both cerebral cortex and hippocampus) on cell death, susceptibility to electroshock-induced convulsions, intrinsic membrane properties, and excitability of cortical and hippocampal neurons.

## Materials and Methods

### Animals

A total of 59 male (offsprings) and 9 female (mothers) rats were involved in the study, including a control group (control rats, C, n = 31) and rats exposed to prenatal hypoxia (hypoxic rats, H, n = 28). Animals were housed under standard conditions at 21 ± 1°C with a 12 h light/dark cycle with free access to food and water. All animal procedures followed ethical principles as stated in the EU Directive 2010/63/EU for animal experiments and the Bioethics Committee of IEPHB RAS (Sechenov Institute of Evolutionary Physiology and Biochemistry of the Russian Academy of Sciences).

### Prenatal Hypoxia Procedure

Female 3–5-month-old Wistar rats were placed with males for mating and every morning vaginal smears were examined until sperm was detected. This day was designated as day 0 of pregnancy. Pregnant females were divided into the Control group (*n* = 4) and the Hypoxia group (*n* = 5). Females from the Hypoxia group on the 14th day of gestation (E14) were exposed to acute normobaric hypoxia in a 100-L chamber where the oxygen level was decreased from 20 to 7% during 15 min and maintained at this level for 3 h (CO_2_ <0.2%, temperature +22°C). Rats from the control group were placed in the same chamber for 3 h, but with normal oxygen levels. The study was performed on the offspring of the rats from these groups. The day of birth was considered as P0.

### Tissue Preparation for Microscopy

**FIGURE 1:**
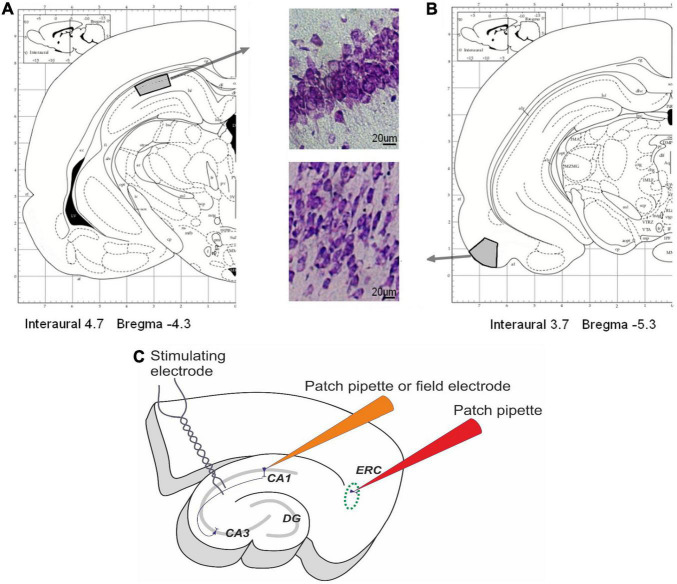
Areas of interest in rat pup brains. **(A**,**B)** The areas of interest in the dorsal hippocampus **(A)** and entorhinal cortex **(B)** for histological investigations. Modified from Paxinos and Watson (2007). **(C)** The scheme of the field potential and whole-cell patch-clamp recordings. Field postsynaptic potentials (fPSPs) and population spikes (PSs) were recorded from CA1 stratum radiatum using glass microelectrodes. Afferent fibers were electrically stimulated by the bipolar electrodes placed in the Schaffer collaterals in the stratum radiatum at the CA1–CA2 border. The membrane properties of pyramidal cells in the deep layers of the medial entorhinal cortex and the CA1 area in the hippocampus were studied using the whole-cell patch-clamp method.

Microscopy analysis of neural tissue structures was performed in control (*n* = 8) and hypoxic (*n* = 9) rats on P20. Rats were anesthetized by Zoletil (Valdepharm, Carros, France, 100 mg/kg of body weight, *i.p.*), and transcardial perfusion with 4% paraformaldehyde in a phosphate buffer (PBS, 4°C, pH 7.4) was performed, then brains were removed and fixed in the same solution for 20 h at 4°C. The 20-μm-thick coronal brain sections were cut using a Leica CM 1510S cryostat (Leica Microsystems, Heidelberg, Germany). For analysis, the sections of the dorsal hippocampus (3.5–5.5 mm) from Bregma and EC (5.3–6.5 mm) were selected (Paxinos and Watson, 2007). A total of eight sections per animal were analyzed. The areas of interest are shown in Figures 1A,B. Brain slices were used to label the neuronal (NeuN protein) and glial (GFAP for astroglia, Iba1 for microglia) marker proteins in the cells.

### Immunochemistry

A sequence of 20-μm slices in the frontal plane was randomly selected and used for immunolabeling by the protocol specified earlier (Shcherbitskaia et al., 2021). Slices were incubated overnight at 25°C in PBS containing 2% bovine serum albumin, 0.3% Triton X-100 (Merck, Darmstadt, Germany), and one of three antibodies: rabbit monoclonal anti-NeuN (ab177487; Abcam, Bristol, United Kingdom; dilution 1:1,000), rabbit polyclonal anti-GFAP (glial fibrillary acidic protein, ab7260, Abcam, 1:200) or rabbit anti-Iba1 (ionized calcium-binding adapter molecule, ab178846, Abcam, 1:100) antibody. After thorough rinsing, the sections were incubated for 1 h at 25°C with secondary antibodies against rabbit IgG: FITC-conjugated (ab97050, Abcam, 1:200) or PE-conjugated (ab7007, Abcam, 1:200) diluted in the blocking serum (2% of BSA and 0.01% Triton X100 in PBS, pH 7.4). Microscopy was performed using a Leica DMR microscope connected to a Leica TCS SP5 confocal scanner (Leica Microsystems, Germany). A 488 nm wavelength He/Ar laser was used for excitation of FITC and PE. Emissions from the FITC, PE were observed in the 496–537 nm and 652–690 nm wavelengths, respectively. The brightness of the cell bodies was measured using the VideoTesT—Master Morphology software program (Video TesT, Russia). A cell was considered immunopositive if the signal exceeded 250% of the background. Nine slices per animal were stained without primary antibodies for the negative control. No traces of non-specific immunoreactivity were observed. Cells of the medial part of the EC were analyzed in a 400 μm wide section including all cortical layers. Hippocampal cells were analyzed in a 400 μm wide section in the lateral part of the CA1 area, including layers *st. oriens*, *st. pyramidale* and *st. radiatum-moleculare*. The numbers of NeuN-positive neurons, GFAP-positive astrocytes, and Iba1-positive microglial cells were counted at the same areas for each brain slice.

### Brain Slice Preparation for Electrophysiological Experiments

The electrophysiological experiments were performed using P21–25 rats (15 control and 11 hypoxic). After decapitation, the brain was quickly removed and placed into a cold (4°C) oxygenated (95% O_2_/5% CO_2_) artificial cerebrospinal fluid (ACSF) containing (in mM): 126 NaCl, 24 NaHCO_3_, 2.5 KCl, 2 CaCl_2_, 1.25 NaH_2_PO_4_, 1 MgSO_4_, and 10 dextrose. Slice preparation was done as previously described (Amakhin et al., 2016; Postnikova et al., 2019). Horizontal brain slices (400 μm) containing the dorsal hippocampus and the adjacent cortical areas, including the EC, were prepared with a vibratome HM 650V (Microm International, Walldorf, Germany). After that, slices were allowed to recover in oxygenated ACSF at 35°C for an hour before electrophysiological experiments.

### Local Field Potential Recording

After incubation, the slices were placed in a recording chamber and perfused with a constant flow of ACSF at a rate of 7 mL/min at room temperature. Field postsynaptic potentials (fPSPs) and population spikes (PSs) were recorded from CA1 stratum radiatum and stratum pyramidale (Figure 1C) accordingly using glass microelectrodes (0.2–1.0 MΩ) as described in detail previously (Postnikova et al., 2019). Afferent fibers were electrically stimulated at a range of current intensities (25–400 mA) by bipolar electrodes placed in the stratum radiatum at the CA1–CA2 border. Responses were registered using a Model 1800 amplifier (A-M Systems, Carlsborg, WA, United States) and were digitized and recorded to a personal computer using NI USB-6211 A/D converter (National Instruments, Austin, TX, United States) and WinWCP v5 software (University of Strathclyde, United Kingdom).

### Whole-Cell Patch-Clamp

Principal neurons in the deep layers of the medial EC or CA1 hippocampus (Figure 1A) were visualized using a Zeiss Axioscop 2 microscope (Zeiss, Jena, Germany) equipped with differential interference contrast optics and a video camera, Grasshopper 3 GS3-U3-23S6M-C (FLIR Integrated Imaging Solutions Inc., Boston, MA, United States). Patch electrodes (3–4 MΩ) were pulled from borosilicate glass capillaries (Sutter Instrument, Novato, CA, United States). A potassium gluconate-based pipette solution (composition in mM: 135 K-Gluconate, 10 NaCl, 5 EGTA, 10 HEPES, 4 ATP-Mg, and 0.3 GTP; pH adjusted to 7.25 with KOH) was used. Whole-cell current-clamp recordings were performed using a Model 2400 (AM-Systems) patch-clamp amplifier and an NI USB-6343 A/D converter (National Instruments) using WinWCP v5 software. The data were sampled at 33 kHz. In all cells included in the sample, access resistance was less than 25 MΩ and remained stable (≤15% increase) across the experiment. Series resistance was evaluated in voltage-clamp mode and compensated for offline numerically. The liquid junction potential was not compensated.

Electrophysiological properties of neurons were assessed from the voltage responses to the series of 1500-ms hyperpolarizing and depolarizing current steps with 10–25 pA increments (Smirnova et al., 2018; Postnikova et al., 2019; Ergina et al., 2021) using custom scripts written in Wolfram Mathematica 12 (Wolfram Research, United States). EC pyramidal neurons were additionally identified by the regular spiking pattern in response to current injection, small afterhyperpolarization, and low steady-state firing frequency (20–40 Hz with 150 pA current injection).

The resting membrane potential (V_*rest*_; in mV) was measured as an averaged potential before the current step application. Membrane input resistance (R_*i*_; in MΩ) was estimated as the slope of the voltage-current (V–I) curve in the interval from –50 to +25 pA. The membrane time constant (τ_membrane_; in ms) was estimated by fitting a single exponential function to the voltage transient induced by the –25 pA current step. Rheobase current (I_*rh*_) refers to the minimum current inducing at least one spike during the step application.

The firing rate-current (f/I) curves were used to describe the firing properties of neurons. The firing rate was estimated as the number of action potentials per 1.5 s current step. The rising part of the f/I curve was fitted with a sigmoidal Gompertz function using Wolfram Mathematica 12:


(1) $$f=f_{m\,a\,x}\,e^{-e^{\left(-k\,\left(I-I_{i\,n\,f\,l}\right)\right)}}$$

where *f*_*max*_ is an asymptote of maximum action potential frequency (in Hz); *e* is Euler’s Number (e = 2.71828.); *k*is a positive number that determines the slope of the curve; *I*_*infl*_ is a value of current (in pA) at which the maximum slope of the curve is observed (inflection current); the maximum slope of the curve (in 1/nA) was calculated as *k*/*e*.

In order to normalize the parameters of f/I curve to the values of V_*rest*_ and R_*i*_, the values of the injected currents (I) were recalculated to the interpolated membrane voltage (U) using the following equation:


(2) $$U=V_{r\,e\,s\,t}+I^{*}\,\,R_{i}\,$$

### Dosed Electroshock

On P20, control (*n* = 8) and hypoxic rats (*n* = 8) were used for dosed electroshock experiments (Peterchev et al., 2010). Susceptibility to electroconvulsive shock (ECS) was tested in seven experiments carried out at one or two-day intervals. All experiments were performed from 11 to 12 a.m. ECS was administered by a pulse generator (ECT Unit 57800; Ugo Basile, Comerio, Italy) through ear-clip electrodes. The following parameters were used: frequency—100 Hz, pulse width 0.7 ms, shock duration 1.5 s (70 pulses for one trial), the current was increased from 10 to 70 mA by 10 mA in each subsequent experiment.

Seizure severity was estimated in each experiment according to the modified Racine scale (Racine, 1972): 1—no seizures; 2—wild running; 3—clonic seizures; 4—tonic seizures without hindlimb extension; 5—tonic seizures with full hindlimb extension.

### Statistical Analysis

The statistical analysis was performed using the Statistica 8.0 (StatSoft, United States) and OriginPro 8 (OriginLab Corporation, United States) software. Dixon’s Q-test (at the 95% confidence level) was used to reject outliers. The normality of the data was tested using the Kolmogorov–Smirnov test. The Levene test was used to verify the equality of variances. The Student’s *t*-test was used for mean comparison for data with a normal distribution and passed an equal variance test. The Welch test was used if the data passed the normality test but failed the equal variance test. Mann–Whitney rank sum test used for the data was unable to pass the normality test. The differences were considered significant at *p* ≤ 0.05. Normally distributed data are presented as mean ± standard error of the mean. The data that failed the normality test are presented as mean and quartiles (25 and 75%).

## Results

### Prenatal Hypoxia Leads to a Neuronal Loss in the EC but Not Hippocampus of Young Rats

**FIGURE 2:**
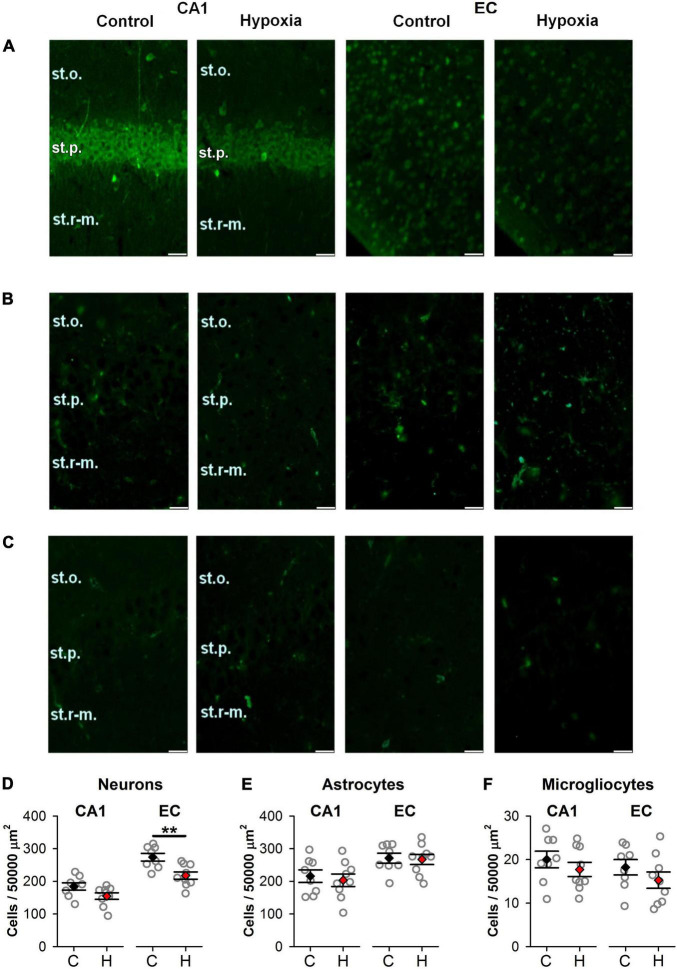
The effect of prenatal hypoxia on the number of neurons and glial cells in the CA1 area of the dorsal hippocampus and entorhinal cortex (EC). **(A–C)** Micrographs of the deep layers of the EC and st. pyramidale of CA1 area of hippocampus in control and “hypoxic” rats. Immunofluorescence (green) of neuronal marker protein NeuN **(A)**, astrocytic marker GFAP **(B)**, and microglia marker Iba1 **(C)**. st.p, stratum pyramidale; st.o, stratum oriens; st.r-m, stratum radiatum-moleculare. Scale bar—50 μm. **(D–F)** The average number of cells in the CA1 area (CA1) of the dorsal hippocampus and EC (EC): NeuN-positive neurons **(D)**, GFAP-positive **(E)**, and Iba1-positive **(F)** glial cells in control (*n* = 8) and hypoxic rats (*n* = 9). Values are presented as mean ± standard error of the mean. The difference between hypoxic and control (black) groups was determined by Student’s *t*-test (**P < 0.01).

We counted the number of neurons, astrocytes, and microglia in the EC and CA1 area of the hippocampus in rats of control and hypoxic groups on P20, using specific markers for these cell types (Figure 2). In the EC of rats subjected to hypoxia, the number of viable neurons expressing NeuN protein was decreased to 79.2% of the control level (Student’s *t*-test, t = 3.5, *P* = 0.003; Figures 2A,D). However, no significant differences between groups were found in the pyramidal layer of the CA1 hippocampus area (t = 1.96, *P* = 0.07; Figure 2D).

In addition, no significant changes in the number of glial cells were observed neither in the EC nor in the CA1 area of hypoxic rats. The average quantity of astrocyte (t = 0.19, *P* = 0.85 for EC and t = 0.46, *P* = 0.65 for CA1; Figures 2B,E) and microglia cells (t = 1.15, *P* = 0.27 for EC and t = 0.92, *P* = 0.37 for CA1; Figures 2C,F) were the same as in control. Thus, we conclude that the EC is more vulnerable to hypoxia on E14 than the CA1 area of the hippocampus.

### Excitatory Postsynaptic Potential-Spike Coupling Was Disturbed After Prenatal Hypoxia

**FIGURE 3:**
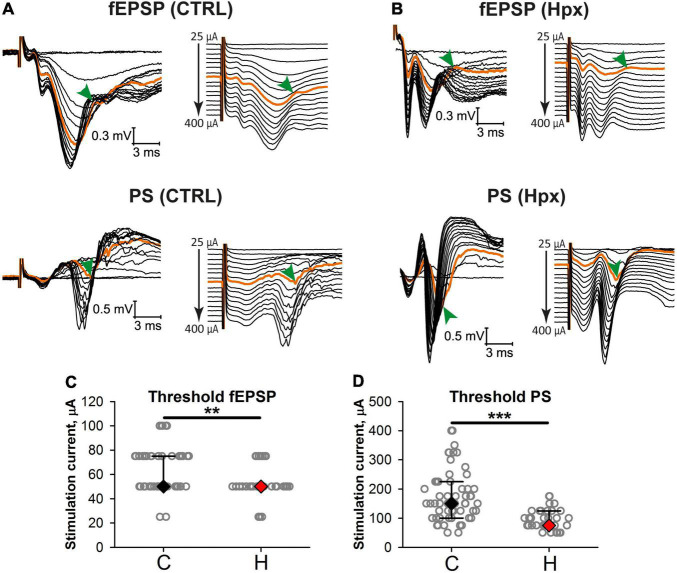
Excitatory postsynaptic potential-spike coupling is altered in rats after prenatal hypoxia. **(A,B)** Representative examples of simultaneous recordings of evoked responses in *stratum radiatum* (top) and *stratum pyramidale* (bottom) to orthodromic stimulation of increasing intensity in slices from the control **(A)** and hypoxic **(B)** groups. On the right, the same recordings are shown with the shift. Orange traces are the traces with the appearance of a population spike (arrowhead). **(C,D)** The threshold current inducing fEPSPs **(C)** and population spikes **(D)** in slices from the control and rats after prenatal hypoxia. **P < 0.01, ***P < 0.001—the difference between the control and hypoxic groups (Mann–Whitney test).

**FIGURE 4:**
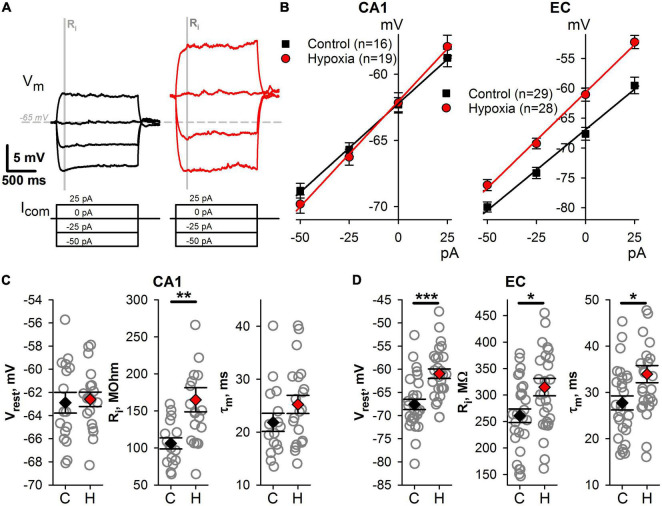
Changes of intrinsic membrane properties of CA1 and EC neurons after prenatal hypoxia. **(A)** A representative example of subthreshold responses to current steps from –50 to +25 pA. The gray bar indicates the time interval (50 ms) which was used to obtain averaged values of membrane potential for V–I relationships. **(B)** The averaged V–I relationships of hippocampal (left panel) and cortical (right panel) neurons in the control group and the group after prenatal hypoxia. **(C,D)** Plots demonstrating the average values of subthreshold parameters of CA1 **(C)** and EC **(D)** neurons in control rats (C) and hypoxic rats (H). Asterisks indicate a significant difference between groups according to an appropriate statistical test. *P < 0.05, **P < 0.01, ***P < 0.001.

Next, we investigated whether the postsynaptic potential-spike coupling was disturbed in rats subjected to prenatal hypoxia. For this purpose, we studied how the amplitude of fPSPs changes with increasing intensity of stimulation current and at what level of stimulation PSs appear in control (*n* = 57 brain slices) and hypoxic (*n* = 37) groups (Figure 4). In most experiments, the threshold current inducing fPSPs was significantly lower in the hypoxic group than in controls (*P* < 0.01, *t*-test, Figures 3A,C). In addition, the threshold of the appearance of a population spike was also lower (*P* < 0.001, *t*-test, Figures 3B,D, orange traces). These findings indicate that the CA1 neuronal network excitability increased after prenatal hypoxia.

### Altered Biophysical Properties of Hippocampal and Cortical Neurons Result in Their Increased Excitability

Next, we compared the subthreshold membrane properties of neurons, including resting membrane potential (V_*r*_), input resistance (R_*i*_), and the membrane time constant (τ) (Figure 4). In hypoxic rats, CA1 pyramidal neurons exhibit significantly larger R_*i*_ (Figures 4B,C, *n* = 19 neurons, 165 ± 16 MΩ) than those in the control group (*n* = 16, 109 ± 7 MΩ, *t*-test, P < 0.01). However, no changes were detected in other subthreshold membrane properties of CA1 neurons. The hypoxia-induced changes of subthreshold properties in EC neurons were more pronounced (Figures 4B,D). Three characteristics under consideration were altered in hypoxic rats. Neurons were more depolarized at rest (control: V_*rest*_ = –67.6 ± 1.1 mV, *n* = 29 vs. hypoxic: –61.0 ± 1.0 mV, *n* = 29, *t*-test, *P* < 0.001), had larger R_*i*_ (control: 261 ± 13 MΩ vs. hypoxia: 315 ± 16 MΩ, *P* < 0.05) and τ (control: 28 ± 2 ms vs. hypoxic: 34 ± 2 ms *P* < 0.05). The increase of the τ can be the result of the increased R_*i*_, as the τ/R_*i*_ ratio was identical in control (111 ± 5 pF) and hypoxic (107 ± 3 pF, *t*-test, *P* = 0.53) groups.

**FIGURE 5:**
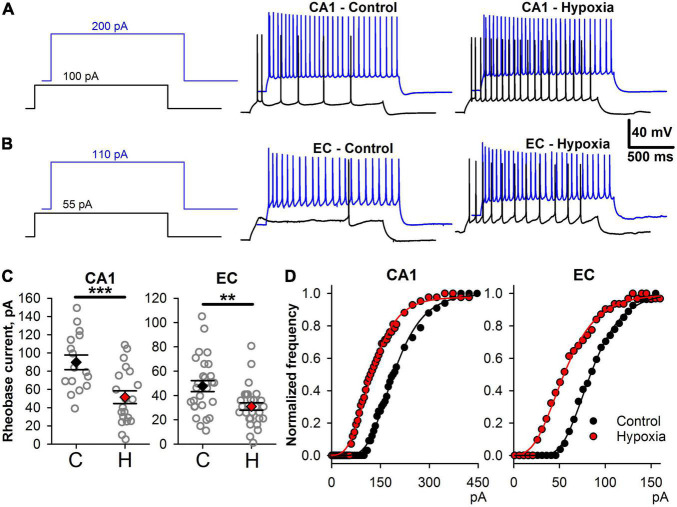
Changes in firing properties of CA1 and EC neurons after prenatal hypoxia. **(A)** The voltage responses to 100 and 200 pA current steps in two representative CA1 neurons from control (CA1-Control) and hypoxic rats (CA1-Hypoxia). **(B)** The voltage responses to 55 and 110 pA current steps in two representative EC neurons from control (EC-Control) and hypoxic rats (EC-Hypoxia). **(C)** Plots demonstrating the average values of rheobase current parameters of CA1-neurons (left) and EC-neurons (right) in control (C) and hypoxic rats (H). Asterisks indicate a significant difference between groups according to an appropriate statistical test. **P < 0.01, ***P < 0.001). **(D)** Representative examples of f/I curves for control (black) and post-hypoxia (red) neurons in CA1 and EC. The data were fitted with the Gompertz function (Eq. 1, thin solid lines).

Changes in intrinsic neuronal properties can disturb the transduction of synaptic input in neuronal firing (Beck and Yaari, 2008). Therefore, we investigated how prenatal hypoxia affects the ability of neurons to transform a constant depolarizing current into action potential generation (Figures 5A,B). In both brain regions, I_*rh*_ value was significantly lower in the hypoxic group (Figure 5C; CA1 neurons, control: 90 ± 8 pA, *n* = 16 *vs.* hypoxia: 51 ± 7 pA, *n* = 20; *t*-test, *P* < 0.001; EC neurons, control: 48 ± 5 pA, *n* = 27 *vs.* hypoxia: 31 ± 3 pA, *n* = 28, *t*-test, *P* < 0.01). These results show that a much smaller current is required to induce AP in neurons in both brain regions of rats subjected to hypoxia.

**FIGURE 6:**
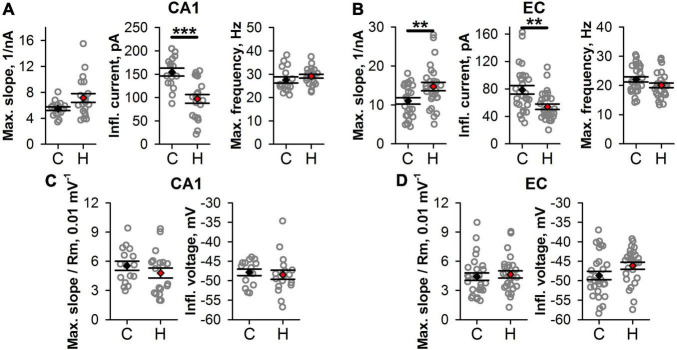
Changes in firing properties of CA1 and EC neurons after prenatal hypoxia. **(A,B)** The graphs show average values of the parameters of f/I curves in CA1 **(A)** and EC **(B)**. **(C,D)** The graphs show the average parameters of the normalized f/I curves. No significant differences between groups are observed after normalization. Asterisks indicate a significant difference between groups according to an appropriate statistical test. **P < 0.01, ***P < 0.001.

Next, we investigated if the shape of f/I relationships was altered in hypoxic rats. We fitted the rising parts of the f/I curves with Eq. 1 (Figure 5D) and compared the obtained parameters in control and “hypoxic” rats. In CA1 pyramidal cells (Figure 6A), only the average value of I_*infl*_ decreased by 37% (control: 154 ± 8 pA, *n* = 16, vs. hypoxic: 97 ± 9 pA, *n* = 20, *P* < 0.001, *t*-test). In EC (Figure 6B), the maximal slope of f/I curve increased by 36% (control: 11 ± 1 nA^–1^, *n* = 27, vs. hypoxic: 15 ± 1 nA^–1^, n = 27, *P* < 0.01, *t*-test), while the inflection current decreased by 32% (control: 79 ± 6 pA, *n* = 28 vs. hypoxic: 54 ± 4 pA, *n* = 28, *P* < 0.01, *t*-test). No significant changes were detected in the maximal firing frequency both in CA1 and EC.

Taken together, these results indicate that in hypoxic rats, the f/I relationships were shifted to the lower values of current compared to those obtained for the control animals. More noticeable changes of f/I curve parameters were observed in EC, as compared to CA1.

To investigate whether the shift of the f/I curve was dependent on detected changes in R_i_ and V_rest_, for each curve, the values of the injected currents were adjusted using Eq. 2. Both in CA1 and EC, no significant changes were detected in the maximal normalized slope and normalized inflection current (Figures 6C,D). This finding indicated that the shift in f/I curves after SE was mainly attributed to subthreshold neuronal properties changes.

### Effects of Prenatal Hypoxia Exposure on Seizure Threshold in Response to Dosed Electroshock

**FIGURE 7:**
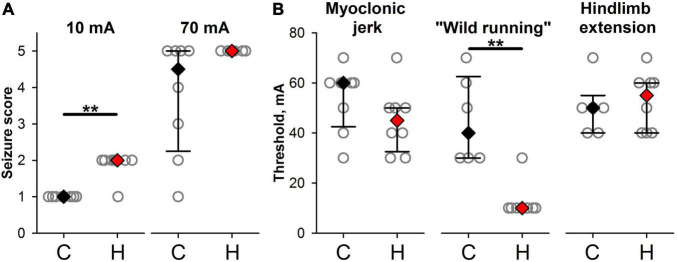
Effects of prenatal hypoxia exposure on seizure reactions in response to dosed electroshock. **(A)** The seizure scores in response to minimal and maximal ECS used in the experiment in control (C) and hypoxic animals (H). **(B)** The threshold amplitudes of electroshock inducing convulsions of different types. ** The difference between the groups at *P* < 0.01 according to the Mann–Whitney test.

As the cortical neurons in hypoxic rats appeared to be more excitable than in the control group, this increased excitability might lead to the increased susceptibility of these animals to the experimentally induced seizures. We investigated the effect of prenatal hypoxia exposure on the seizure threshold in response to the dosed ECS. The hypoxic rats were more vulnerable to ECS than the control animals. The minimal ECS used in the experiment (10 mA) did not produce a visible reaction in control rats (the seizure score was equal to 1 in all animals) but induced wild running in most rats from the hypoxic group (in 7 out of 8 animals the seizure score was equal to 2, Mann–Whitney test, *P* = 0.0014, Figure 7A). The largest current intensity used in the present study (70 mA) produces all types of seizures in control rats, while in hypoxic rats, only tonic seizures with hindlimbs extension were observed [control: 4.5 (2.25;5) points vs. hypoxic: 5 points in all animals, Figure 7A].

Prenatal hypoxia decreases the thresholds for wild running (Figure 7B, control: 45 ± 7 mA, hypoxic: 13 ± 3 mA, Mann–Whitney test, *P* < 0.01).

## Discussion

This study describes the long-term effects of maternal hypoxia on the intrinsic electrical properties and excitability of neurons in the EC and hippocampus. We observed a decrease of threshold current inducing fEPSPs in slices from the hypoxic rats after prenatal hypoxia and a reduced threshold of the appearance of population spike that indicates increased excitability of CA1 neurons after prenatal hypoxia. Integration of synaptic excitation to generate an action potential (excitatory postsynaptic potential-spike coupling or E-S coupling) determines the neuronal output (Daoudal et al., 2002).

The current study demonstrates profound alterations in intrinsic membrane properties of cortical and hippocampal neurons of rats subjected to hypoxia, leading to an increase of their excitability. The neuronal intrinsic membrane properties are derived from the activity of multiple types of ion channels (Amarillo et al., 2014), the expression or function of which can be altered in hypoxic rats. The alterations of membrane biophysical properties were previously reported in several studies that utilized *in vitro* hypoxia models in rat brain slices. A significant depolarization of CA1 pyramidal neurons, accompanied by the increased excitability and downregulation of hyperpolarization-activated currents, was observed during a period of hypoxia *in vitro* (Zanelli et al., 2015). The combination of hypoxia and inflammation reduced the excitability of hippocampal neurons in rat brain slices. In contrast, reoxygenation caused their hyperexcitability, attributable to the regulation of hyperpolarization-activated currents and changes in the input resistance (Yang et al., 2018). Transient hyperexcitability and increased input resistance were observed in a fraction of neocortical neurons on reoxygenation following hypoxia (Luhmann and Heinemann, 1992).

Hypoxia *in vitro* also induced the negative modulation of several voltage-gated potassium channels (Liu et al., 2014; Yang et al., 2021), which strongly affected neuronal excitability. The downregulation of A-type potassium currents has also been reported in the hippocampus after seizure-inducing hypoxia in neonatal rats (Peng et al., 2013). In addition, several reports indicate that neonatal hypoxia can affect intracellular Ca^2+^ signaling (Silver and Erecińska, 1990; Sanchez et al., 2001; Lippman-Bell et al., 2016), potentially resulting in altered Ca^2+^-dependent modulation of various ion channels (Nelson et al., 2003; Segal, 2018). Thus, the intrinsic membrane properties can be quite sensitive to hypoxia, which provides an opportunity for a significant modulation of neuronal excitability.

It can also be suggested that alterations in neurons’ biophysical properties may be due to morphological reorganization induced by prenatal hypoxia. We observed a decrease in the number of NeuN-positive neuronal cells in EC of hypoxic rats compared to control, suggesting neuronal death, but there was no gliosis associated with the neuroinflammation process. The data about neuronal death presented here aligns with our previous observations for the parietal cortical area (Vasilev et al., 2016) and the result of another study (Vetrovoy et al., 2021).

However, in our previous works focused on the study of structural abnormalities in the parietal cortex (Vasil’ev et al., 2008), EC (Vasilev et al., 2021), and hippocampus (Zhuravin et al., 2009), we analyzed Nissl stained brain slices. The data concerning the distribution of the NeuN neuronal marker and glia cell markers in the EC and CA1 of the hippocampus of hypoxic rats presented in this study suggest that prenatal hypoxia leads to the death of neurons in EC but not to the activation of glial cells. The molecular mechanisms of neuronal death in hypoxic rats are not known. The absence of detectable gliosis suggests that neuroinflammation mechanisms do not significantly affect hypoxia-induced cell loss. Several reports have suggested that caspase-associated apoptosis could be involved (Vasil’ev et al., 2008; Kozlova et al., 2015), but autophagy (Descloux et al., 2015) and excitotoxic (Vetrovoy et al., 2021) mechanisms are also possible. In this study, we showed an increase in the excitability of the neurons that particularly suggests the involvement of the excitotoxic mechanism.

Another possible mechanism of the increased excitation in the cortical neuronal circuitry may be associated with dysfunction of the inhibitory system. For example, in mice subjected to hypoxia on E13–E15, there is a loss of cortical GABAergic neurons, and the population of the calbindin-positive interneurons is the most affected one (Nisimov et al., 2018). The dysfunction of some interneuron populations might disturb the balance between excitation and inhibition. The data obtained in this study suggest that prenatal hypoxia affects intrinsic mechanisms of neuronal excitation. However, the present study does not exclude potential changes in the inhibition mechanism, and further studies are needed to clarify this phenomenon.

It can also be concluded that the EC is more vulnerable to the destructive action of hypoxia on E14 than the CA1 area of the hippocampus. Such a difference in vulnerability might be predictable considering that hypoxia on E14 specifically disturbs radial neuroblast migration in cortical areas, including the entorhinal cortex (Vasilev et al., 2016, 2021). However, it does not directly affect the dorsal hippocampus that emerges later in embryogenesis (Rice and Barone, 2000). Hypoxia on E17 also disturbs neuroblast migration into the hippocampus leading to neuronal death after birth (Golan et al., 2009). Thus, the differences between EC and CA1 in the changes of the neuronal cell numbers can be associated with differences in critical periods for these two brain regions.

The generation and migration of the hippocampus progenitor cells in rats occur not earlier than on E16. Thus, the hippocampus cannot be affected directly by the hypoxia on E14. The most probable cause of the hippocampus vulnerabilities might be based on the intimate connection between the hippocampus and brain cortical areas (e.g., prefrontal cortex, entorhinal cortex). The hypoxia on E14 disrupts the formation of the entorhinal cortex (Vasilev et al., 2021), especially the projection neurons forming the temporoammonic pathway directed to the CA1 area. We showed earlier a decrease in the number of synapses formed by synaptopodin-positive labile dendritic spines in the CA1 area of the hippocampus of P20–30 rats subjected to prenatal hypoxia on E14 (Zhuravin et al., 2019). A similar decrease was shown on the model of deafferentation of apical dendrites in hippocampus pyramidal neurons induced by the experimental lesion in the entorhinal cortical area (Vlachos et al., 2013). Thus, the hypoxia-induced neuronal death in the entorhinal cortical area might lead to the disruption of the hippocampal afferentation affecting neuronal functions in the CA1 area.

Structural and biophysical changes in the neuronal circuitries may lead to hyperexcitability and some neurological diseases. We noted that hypoxic rats were more sensitive to electroshock. Clonic seizures are initiated in limbic structures, the hippocampus, dentate gyrus, amygdala, piriform cortex, perirhinal cortex, EC, and nucleus accumbens, while tonic seizure—in the lower tubercle (André et al., 2002). Thus, the differences between hypoxia and control groups in the ECS experiment might be associated with hypoxia-induced increased excitability of neurons and ultrastructural reorganization in EC and hippocampus.

The data obtained strongly suggest that hypoxia at the time of generation of the pyramidal cortical neurons (E14) leads to the altered excitation properties of neuronal cells in CA1 of hippocampus and EC of young rat brain and is accompanied by the increased cell death in EC. The changes of intrinsic parameters in EC neurons are more prominent than in hippocampal neurons. The molecular mechanisms of the increase in the excitability of the glutamatergic neurons after maternal hypoxia should be investigated, as the disturbances in forebrain development might increase the risk of epilepsy in later life as is known for perinatal hypoxia.

## Data Availability Statement

The raw data supporting the conclusions of this article will be made available by the authors, without undue reservation.

## Ethics Statement

The animal study was reviewed and approved by Bioethics Committee of IEPHB RAS Sechenov Institute of Evolutionary Physiology and Biochemistry, Russian Academy of Sciences, St. Petersburg, Russia.

## Author Contributions

DV, DK, and AZ conceived and designed the experiments. DA, ES, and TP performed the electrophysiological research. DV and NT provided the histochemical part. DK and ND obtained and analyzed the dosed electroshock data. DV, DA, DK, and AZ wrote the manuscript. All authors revised and approved the final manuscript.

## Conflict of Interest

The authors declare that the research was conducted in the absence of any commercial or financial relationships that could be construed as a potential conflict of interest.

## Publisher’s Note

All claims expressed in this article are solely those of the authors and do not necessarily represent those of their affiliated organizations, or those of the publisher, the editors and the reviewers. Any product that may be evaluated in this article, or claim that may be made by its manufacturer, is not guaranteed or endorsed by the publisher.
